# 2-(1,2,3,4-Tetra­hydro­phenanthren-1-yl­idene)malononitrile

**DOI:** 10.1107/S1600536809023988

**Published:** 2009-06-27

**Authors:** George B. Ettenger, Brian Wesley Williams, Daniel Brillhart, Margaret E. Kastner

**Affiliations:** a Department of Chemistry, Bucknell University, Lewisburg, PA 17837, USA

## Abstract

In the title complex, C_17_H_12_N_2_, the non-aromatic six-membered ring adopts an envelope conformation. The dihedral angle between the eight-membered plane containing the malononitrile group and the aromatic system is 25.88 (4)°. The distance from the central C atom of the malononitrile group to the centroid of the *n*-glide-related distal aromatic ring is 3.66 Å, suggesting π–π inter­actions.

## Related literature

For a related structure, see: Nesterov *et al.* (2001 ▶). For solvatochromism in 2-(naphthalen-1-ylmethyl­ene)malononitrile and related systems, see: Katritzky *et al.* (1991 ▶). For a description of the Cambridge Structural Database, see: Allen *et al.* (2002 ▶);
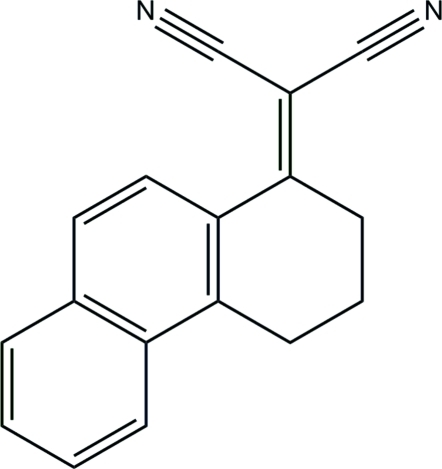


## Experimental

### 

#### Crystal data

C_17_H_12_N_2_
*M* *_r_* = 244.29
Monoclinic, 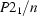

*a* = 7.3990 (9) Å
*b* = 16.190 (3) Å
*c* = 10.4570 (13) Å
β = 93.016 (7)°
*V* = 1250.9 (3) Å^3^
*Z* = 4
Mo *K*α radiation
μ = 0.08 mm^−1^
*T* = 293 K
0.5 × 0.2 × 0.2 mm

#### Data collection

Bruker P4 diffractometer
Absorption correction: none
4319 measured reflections
3151 independent reflections
1297 reflections with *I* > 2σ(*I*)
*R*_int_ = 0.044
3 standard reflections every 97 reflections intensity decay: none

#### Refinement

*R*[*F* ^2^ > 2σ(*F* ^2^)] = 0.072
*wR*(*F* ^2^) = 0.176
*S* = 0.98
3151 reflections
173 parameters
H-atom parameters constrained
Δρ_max_ = 0.22 e Å^−3^
Δρ_min_ = −0.21 e Å^−3^

### 

Data collection: *XSCANS* (Bruker, 1996 ▶); cell refinement: *XSCANS*; data reduction: *XSCANS*; program(s) used to solve structure: *SHELXS97* (Sheldrick, 2008 ▶); program(s) used to refine structure: *SHELXL97* (Sheldrick, 2008 ▶); molecular graphics: *SHELXTL* (Sheldrick, 2008 ▶); software used to prepare material for publication: *SHELXTL*.

## Supplementary Material

Crystal structure: contains datablocks I, global. DOI: 10.1107/S1600536809023988/pv2168sup1.cif

Structure factors: contains datablocks I. DOI: 10.1107/S1600536809023988/pv2168Isup2.hkl

Additional supplementary materials:  crystallographic information; 3D view; checkCIF report

## Figures and Tables

**Fig. 1.**
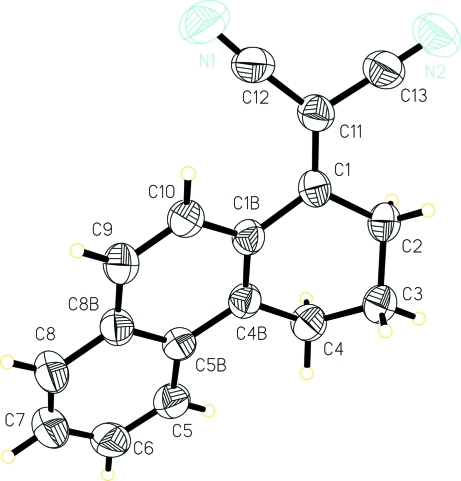
The molecular structure of the title compound, with atom labels and 50% probability displacement ellipsoids for non-H atoms.

**Fig. 2.**
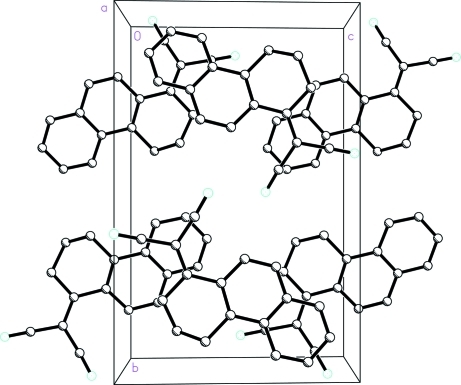
The packing of the title compound, looking at the bc-face showing the overlap of the malononitrile group of one molecule with the distal aromatic ring of an n-glide related molecule.

## supplementary crystallographic information

### Comment

The title complex was prepared and the structure determined as part of a study on solvatochromism by fluorescence emission. Solvatochromism in 2-(naphthalen-1-ylmethylene)malononitrile and related systems (reported as dicyanovinyl substituted aromatics) was attributed to formation of a "twisted intramolecular charge transfer" (TICT) emitting state, involving rotation about a single covalent bond with charge transfer from the aromatic groups to the electrophilic moeity (Katritzky *et al.*, 1991). The structure of 2-(naphthalen-1-ylmethylene)malononitrile (reported as 1,1-dicyano-2-(1-naphthyl)ethylene) has since been reported (Nesterov *et al.*, 2001). The dihdral angle between the 8-atom plane containing the malononitrile group and the 10-atom aromatic system is 41° [based on coordinates reported in the Cambridge Structural Database (Allen *et al.*, 2002) as refcode XINCUS]. This large twist angle may result from the close H—H distance between the ethylene-hydrogen and the *peri*-hydrogen of 2.2 Å.

The study currently underway removes the constraint of a *peri*-hydrogen by looking at 2-(naphthalen-2-ylmethylene)malononitriles. In terms of rotation about the single bond, these compounds are analogous to simple 2-benzylidenemalononitrile derivatives. Forty-nine such compounds are reported in the CSD. In these compounds the dihedral angle between the 8-atom plane containing the malononitrile group and the benzene group ranges from 2° to 27°, with an average of 10 (6) °. The dihedral angle between the 8-atom plane containing the malononitrile group and the 10-atom aromatic system in the title compound is 25.88 (0.04) °.

In the title complex the non-aromatic six-membered ring is constrained by both the fused aromatic ring and the conjugated alkene functional group. The plane C1 C1b C4 C4b makes a dihedral angle of 6.12 (0.07) ° with the aromatic system; the displacements of C2 and C3 from this plane are -0.35 Å and -0.89 Å, respectively, resulting in an envelope conformation.

The packing diagram shown in Fig. 2 shows the overlap of the malononitrile group with the distal aromatic ring of a molecule related by the n-glide (-1/2 + *x*, 1.5 - *y*, -1/2 + *z*). The distance from C11 to the centroid of this ring is 3.66 Å, suggesting π-π interactions.

### Experimental

The title compound was synthesized by mixing 0.24 g (1.2 mmol) 3,4-dihydrophenanthren-1(2*H*)-one with 0.26 g (3.9 mmol) malononitrile and 0.22 g (1.1 mmol) of sodium acetate trihydrate in approximately 20 ml of absolute ethanol. The solution was stirred and refluxed under nitrogen for 13 h. The solution initially is yellow, becoming progressively darker with heating and resulting in a green/yellow precipitate in a brown solution. Solid was then collected by vacuum filtration for a crude yield of 0.15 g. This was then separated by flash chromatography using 20% ethylacetate in hexanes yielding 0.03 g (0.12 mmol, 10% yield). Crystals of the title compound were grown at room temperature by vapor diffusion of ethanol into a dichloromethane solution.

### Refinement

Hydrogen positions were calculated and refined using a riding model using the following C—H distances: methylene 0.97 Å and aromatic 0.93 Å. The isotropic U values for the H atoms were set at 20% above that of the bonded carbon.

### Crystal data

**Table:**

C_17_H_12_N_2_	*F*(000) = 512
*M**_r_* = 244.29	*D*_x_ = 1.297 Mg m^−^^3^
Monoclinic, *P*2_1_/*n*	Mo *K*α radiation, λ = 0.71073 Å
Hall symbol: -P 2yn	Cell parameters from 27 reflections
*a* = 7.3990 (9) Å	θ = 20–25°
*b* = 16.190 (3) Å	µ = 0.08 mm^−^^1^
*c* = 10.4570 (13) Å	*T* = 293 K
β = 93.016 (7)°	Needle, yellow
*V* = 1250.9 (3) Å^3^	0.5 × 0.2 × 0.2 mm
*Z* = 4	

### Data collection

**Table:**

Bruker P4 diffractometer	*R*_int_ = 0.044
Radiation source: fine-focus sealed tube	θ_max_ = 28.5°, θ_min_ = 2.5°
graphite	*h* = −9→1
2θ/ω scans	*k* = −1→21
4319 measured reflections	*l* = −14→14
3151 independent reflections	3 standard reflections every 97 reflections
1297 reflections with *I* > 2σ(*I*)	intensity decay: none

### Refinement

**Table:**

Refinement on *F*^2^	Primary atom site location: structure-invariant direct methods
Least-squares matrix: full	Secondary atom site location: difference Fourier map
*R*[*F*^2^ > 2σ(*F*^2^)] = 0.072	Hydrogen site location: inferred from neighbouring sites
*wR*(*F*^2^) = 0.176	H-atom parameters constrained
*S* = 0.98	*w* = 1/[σ^2^(*F*_o_^2^) + (0.0683*P*)^2^] where *P* = (*F*_o_^2^ + 2*F*_c_^2^)/3
3151 reflections	(Δ/σ)_max_ = 0.003
173 parameters	Δρ_max_ = 0.22 e Å^−^^3^
0 restraints	Δρ_min_ = −0.21 e Å^−^^3^

### Special details

**Table:**

Experimental. ^1^H NMR (400 MHz, CDCl_3_): δ 2.207 (2*H*, m), 3.073 (2*H*, t), 3.348 (2*H*,t), 7.626 (2*H*, m), 7.800 (1*H*, d), 8.873(1*H*, dd), 8.102 (1*H*, dd), 8.159 (1*H*, d).^13^C NMR (400 MHz, CDCl_3_): δ 22.444, 25.919, 32.966, 79.691, 113.691,114.153, 123.313, 124.839, 127.220, 127.428, 127.570, 128.842, 123.857,131.357, 135.443, 140.012, 173.573
Geometry. All e.s.d.'s (except the e.s.d. in the dihedral angle between two l.s. planes) are estimated using the full covariance matrix. The cell e.s.d.'s are taken into account individually in the estimation of e.s.d.'s in distances, angles and torsion angles; correlations between e.s.d.'s in cell parameters are only used when they are defined by crystal symmetry. An approximate (isotropic) treatment of cell e.s.d.'s is used for estimating e.s.d.'s involving l.s. planes.
Refinement. Refinement of *F*^2^ against ALL reflections. The weighted *R*-factor *wR* and goodness of fit *S* are based on *F*^2^, conventional *R*-factors *R* are based on *F*, with *F* set to zero for negative *F*^2^. The threshold expression of *F*^2^ > σ(*F*^2^) is used only for calculating *R*-factors(gt) *etc*. and is not relevant to the choice of reflections for refinement. *R*-factors based on *F*^2^ are statistically about twice as large as those based on *F*, and *R*- factors based on ALL data will be even larger.

### Fractional atomic coordinates and isotropic or equivalent isotropic displacement parameters (Å^2^)

**Table:**

	*x*	*y*	*z*	*U*_iso_*/*U*_eq_	
N1	0.2071 (5)	0.50142 (17)	0.3832 (3)	0.0865 (11)	
N2	0.1459 (4)	0.60547 (17)	0.0118 (3)	0.0799 (10)	
C1	0.0989 (4)	0.71078 (16)	0.2975 (2)	0.0385 (7)	
C1B	0.0931 (4)	0.73306 (16)	0.4337 (2)	0.0397 (7)	
C2	0.0720 (4)	0.77843 (16)	0.1988 (2)	0.0457 (7)	
H2A	0.1836	0.7856	0.1553	0.055*	
H2B	−0.0208	0.7613	0.1354	0.055*	
C3	0.0184 (4)	0.86019 (17)	0.2540 (2)	0.0507 (8)	
H3A	−0.1066	0.8577	0.2774	0.061*	
H3B	0.0283	0.9032	0.1901	0.061*	
C4	0.1393 (4)	0.88100 (16)	0.3713 (2)	0.0505 (8)	
H4A	0.2639	0.8853	0.3476	0.061*	
H4B	0.1038	0.9338	0.4059	0.061*	
C4B	0.1234 (4)	0.81471 (16)	0.4709 (2)	0.0408 (7)	
C5	0.1765 (4)	0.91533 (18)	0.6502 (3)	0.0533 (8)	
H5	0.2017	0.9568	0.5923	0.064*	
C5B	0.1328 (4)	0.83533 (17)	0.6048 (2)	0.0416 (7)	
C6	0.1823 (4)	0.9326 (2)	0.7785 (3)	0.0633 (10)	
H6	0.2130	0.9855	0.8067	0.076*	
C7	0.1429 (5)	0.8722 (2)	0.8671 (3)	0.0652 (10)	
H7	0.1442	0.8853	0.9537	0.078*	
C8	0.1028 (4)	0.7944 (2)	0.8277 (3)	0.0583 (9)	
H8	0.0781	0.7543	0.8879	0.070*	
C8B	0.0979 (4)	0.77342 (18)	0.6966 (2)	0.0445 (7)	
C9	0.0578 (4)	0.69297 (17)	0.6539 (2)	0.0479 (8)	
H9	0.0324	0.6525	0.7135	0.057*	
C10	0.0553 (4)	0.67254 (17)	0.5278 (2)	0.0452 (7)	
H10	0.0286	0.6186	0.5026	0.054*	
C11	0.1345 (4)	0.63407 (17)	0.2542 (2)	0.0457 (7)	
C12	0.1745 (5)	0.5615 (2)	0.3295 (3)	0.0573 (9)	
C13	0.1401 (4)	0.61857 (17)	0.1187 (3)	0.0552 (8)	

### Atomic displacement parameters (Å^2^)

**Table:**

	*U*^11^	*U*^22^	*U*^33^	*U*^12^	*U*^13^	*U*^23^
N1	0.129 (3)	0.0574 (17)	0.076 (2)	0.026 (2)	0.028 (2)	0.0168 (16)
N2	0.114 (3)	0.074 (2)	0.0535 (17)	0.005 (2)	0.0163 (17)	−0.0116 (15)
C1	0.0392 (16)	0.0427 (16)	0.0338 (14)	−0.0053 (14)	0.0044 (12)	0.0037 (12)
C1B	0.0420 (17)	0.0416 (16)	0.0358 (15)	0.0017 (14)	0.0051 (12)	0.0059 (12)
C2	0.0520 (19)	0.0472 (17)	0.0379 (15)	−0.0008 (16)	0.0028 (13)	0.0093 (13)
C3	0.063 (2)	0.0458 (17)	0.0430 (15)	0.0042 (16)	0.0012 (15)	0.0085 (14)
C4	0.067 (2)	0.0387 (16)	0.0465 (16)	−0.0031 (16)	0.0056 (15)	0.0064 (13)
C4B	0.0412 (17)	0.0423 (16)	0.0391 (14)	0.0022 (14)	0.0028 (13)	0.0051 (13)
C5	0.061 (2)	0.0454 (17)	0.0531 (18)	0.0064 (16)	−0.0051 (15)	−0.0029 (15)
C5B	0.0403 (17)	0.0437 (16)	0.0407 (15)	0.0057 (14)	0.0011 (13)	−0.0006 (13)
C6	0.075 (3)	0.057 (2)	0.0558 (19)	0.0150 (19)	−0.0113 (18)	−0.0187 (17)
C7	0.075 (2)	0.078 (2)	0.0421 (17)	0.013 (2)	−0.0020 (16)	−0.0074 (18)
C8	0.065 (2)	0.072 (2)	0.0369 (16)	0.0093 (19)	−0.0006 (15)	−0.0003 (16)
C8B	0.0421 (18)	0.0534 (18)	0.0379 (15)	0.0060 (16)	0.0019 (13)	0.0017 (14)
C9	0.0543 (19)	0.0487 (17)	0.0410 (16)	−0.0011 (16)	0.0063 (14)	0.0073 (14)
C10	0.0542 (19)	0.0390 (16)	0.0427 (16)	−0.0006 (15)	0.0053 (14)	0.0043 (13)
C11	0.056 (2)	0.0419 (16)	0.0396 (15)	−0.0025 (15)	0.0080 (14)	0.0013 (14)
C12	0.075 (2)	0.0450 (17)	0.0533 (18)	0.0090 (18)	0.0137 (16)	−0.0025 (16)
C13	0.067 (2)	0.0502 (18)	0.0494 (18)	0.0001 (17)	0.0098 (16)	−0.0040 (15)

### Geometric parameters (Å, °)

**Table:**

N1—C12	1.143 (3)	C5—C6	1.368 (4)
N2—C13	1.140 (3)	C5—C5B	1.411 (4)
C1—C11	1.353 (3)	C5—H5	0.9300
C1—C1B	1.472 (3)	C5B—C8B	1.421 (4)
C1—C2	1.511 (3)	C6—C7	1.388 (4)
C1B—C4B	1.393 (3)	C6—H6	0.9300
C1B—C10	1.427 (3)	C7—C8	1.353 (4)
C2—C3	1.506 (4)	C7—H7	0.9300
C2—H2A	0.9700	C8—C8B	1.411 (4)
C2—H2B	0.9700	C8—H8	0.9300
C3—C4	1.517 (4)	C8B—C9	1.404 (4)
C3—H3A	0.9700	C9—C10	1.359 (3)
C3—H3B	0.9700	C9—H9	0.9300
C4—C4B	1.504 (3)	C10—H10	0.9300
C4—H4A	0.9700	C11—C12	1.436 (4)
C4—H4B	0.9700	C11—C13	1.442 (4)
C4B—C5B	1.438 (3)		
			
C11—C1—C1B	124.4 (2)	C6—C5—H5	119.6
C11—C1—C2	117.2 (2)	C5B—C5—H5	119.6
C1B—C1—C2	118.3 (2)	C5—C5B—C8B	117.8 (2)
C4B—C1B—C10	119.6 (2)	C5—C5B—C4B	122.6 (2)
C4B—C1B—C1	119.4 (2)	C8B—C5B—C4B	119.5 (3)
C10—C1B—C1	121.0 (2)	C5—C6—C7	120.9 (3)
C3—C2—C1	113.7 (2)	C5—C6—H6	119.6
C3—C2—H2A	108.8	C7—C6—H6	119.6
C1—C2—H2A	108.8	C8—C7—C6	120.2 (3)
C3—C2—H2B	108.8	C8—C7—H7	119.9
C1—C2—H2B	108.8	C6—C7—H7	119.9
H2A—C2—H2B	107.7	C7—C8—C8B	121.0 (3)
C2—C3—C4	110.4 (2)	C7—C8—H8	119.5
C2—C3—H3A	109.6	C8B—C8—H8	119.5
C4—C3—H3A	109.6	C9—C8B—C8	121.8 (3)
C2—C3—H3B	109.6	C9—C8B—C5B	118.8 (2)
C4—C3—H3B	109.6	C8—C8B—C5B	119.3 (3)
H3A—C3—H3B	108.1	C10—C9—C8B	121.8 (3)
C4B—C4—C3	109.7 (2)	C10—C9—H9	119.1
C4B—C4—H4A	109.7	C8B—C9—H9	119.1
C3—C4—H4A	109.7	C9—C10—C1B	120.6 (3)
C4B—C4—H4B	109.7	C9—C10—H10	119.7
C3—C4—H4B	109.7	C1B—C10—H10	119.7
H4A—C4—H4B	108.2	C1—C11—C12	127.2 (2)
C1B—C4B—C5B	119.4 (2)	C1—C11—C13	120.4 (2)
C1B—C4B—C4	120.1 (2)	C12—C11—C13	112.4 (2)
C5B—C4B—C4	120.5 (2)	N1—C12—C11	176.2 (3)
C6—C5—C5B	120.8 (3)	N2—C13—C11	179.1 (4)
			
C11—C1—C1B—C4B	155.9 (3)	C4—C4B—C5B—C8B	173.2 (3)
C2—C1—C1B—C4B	−21.1 (4)	C5B—C5—C6—C7	−0.9 (5)
C11—C1—C1B—C10	−24.7 (4)	C5—C6—C7—C8	1.8 (5)
C2—C1—C1B—C10	158.3 (3)	C6—C7—C8—C8B	−0.8 (5)
C11—C1—C2—C3	175.4 (3)	C7—C8—C8B—C9	179.5 (3)
C1B—C1—C2—C3	−7.4 (4)	C7—C8—C8B—C5B	−1.0 (5)
C1—C2—C3—C4	47.2 (3)	C5—C5B—C8B—C9	−178.7 (3)
C2—C3—C4—C4B	−59.5 (3)	C4B—C5B—C8B—C9	1.0 (4)
C10—C1B—C4B—C5B	5.8 (4)	C5—C5B—C8B—C8	1.9 (4)
C1—C1B—C4B—C5B	−174.8 (2)	C4B—C5B—C8B—C8	−178.4 (3)
C10—C1B—C4B—C4	−172.0 (3)	C8—C8B—C9—C10	−179.2 (3)
C1—C1B—C4B—C4	7.3 (4)	C5B—C8B—C9—C10	1.4 (4)
C3—C4—C4B—C1B	32.9 (4)	C8B—C9—C10—C1B	−0.2 (4)
C3—C4—C4B—C5B	−144.9 (3)	C4B—C1B—C10—C9	−3.4 (4)
C6—C5—C5B—C8B	−1.0 (4)	C1—C1B—C10—C9	177.2 (3)
C6—C5—C5B—C4B	179.4 (3)	C1B—C1—C11—C12	−0.7 (5)
C1B—C4B—C5B—C5	175.1 (3)	C2—C1—C11—C12	176.3 (3)
C4—C4B—C5B—C5	−7.1 (4)	C1B—C1—C11—C13	−179.0 (3)
C1B—C4B—C5B—C8B	−4.6 (4)	C2—C1—C11—C13	−1.9 (4)
